# Effects of a Generative AI Virtual Patient on Serious Illness Communication Skills: Randomized Controlled Trial

**DOI:** 10.2196/93034

**Published:** 2026-10-20

**Authors:** Kurtis G Haut, Masum Hasan, Thomas Carroll, Ronald M Epstein, Taylan Sen, Ehsan Hoque

**Affiliations:** 1 Department of Computer Science, University of Rochester, 3013 Wegmans Hall, Rochester, NY, 14627, United States, +1 (585) 275-1351; 2 Department of Medicine, Divisions of General Medicine and Palliative Care, University of Rochester Medical Center, Rochester, NY, United States; 3 Departments of Family Medicine and Medicine, University of Rochester Medical Center, Rochester, NY, United States; 4 Computer and Information Sciences, Niagara University, Niagara Falls, NY, United States

**Keywords:** virtual standardized patients, AI, large language models, generative AI, serious illness communication, palliative care, end-of-life care, randomized controlled trial, medical education, simulation-based training

## Abstract

**Background:**

Serious illness communication (SIC) is a critical skill in medical education, especially in end-of-life care, yet clinicians often lack sufficient opportunities for deliberate practice. Standardized patients (SPs) remain the gold standard for SIC training, but they are limited by cost, availability, and consistency. To support wider access to SIC training, scalable alternatives are needed.

**Objective:**

The aim of this study is to evaluate whether SOPHIE (Standardized Online Patient for Healthcare Interaction Education), an AI-powered virtual patient that provides real-time dialogue and automated feedback, improves SIC skills among clinicians and trainees.

**Methods:**

We conducted a single-blind, parallel-group, randomized controlled trial from June to December 2024 via videoconferencing. Participants were randomized in a 1:1 ratio to the intervention (SOPHIE training) or control (reading module) group. Blinded SP actors and 4 independent raters evaluated outcomes preintervention and postintervention. The intervention group completed 3 interactive SOPHIE modules (Empathize, Be Explicit, and Empower) for 30 minutes, combining dialogue with an interactive virtual patient and automated feedback. Control participants reviewed reading materials covering the same framework for the same duration. The primary outcome was the change in communication skills (Empathize, Be Explicit, and Empower) assessed by SPs and blinded raters using a rubric based on an established clinical communication training framework. Secondary outcomes included self-reported confidence in communication skills.

**Results:**

Fifty-one medical, nursing, and physician assistant students and practicing clinicians (mean age 30.6, SD 13.2 years; women: 40/51, 78.4%) were recruited from academic and clinical settings. Participants were stratified by training level and randomized; all completed the study. Compared with controls, SOPHIE participants demonstrated significantly greater improvement in Empower (β=.11, 95% CI .04-.17; *P*=.002), Be Explicit (β=.08, 95% CI .03-.13; *P*=.002), Empathize (β=.07, 95% CI .01-.13; *P*=.03), and overall communication score (β=.09, 95% CI .04-.14; *P*=.001). Here, β represents the additional pre-to-post improvement in the SOPHIE arm relative to the control arm, estimated from linear mixed effects models. SOPHIE participants also reported higher confidence in their communication skills than controls (mean score of 4.31 vs 3.83 on a 5-point Likert scale; *P*=.009).

**Conclusions:**

Training with an AI-powered virtual patient significantly improved SIC skills and confidence compared with reading modules. Generative AI–based simulations may provide a scalable, accessible, and effective adjunct or alternative to traditional SP training in clinical education.

## Introduction

Effective serious illness communication (SIC) in end-of-life care is a universally recognized gap in medical training [1-4]. These are high-stakes conversations in which clinicians must navigate weighty issues, and poor communication has been linked to prognostic misunderstanding [5], perceived lack of support [6], overtreatment [7-9], higher health care costs [10,11], and clinician burnout and malpractice risk [12-14]. National consensus statements on communication in oncology settings prioritize improving SIC [15,16].

Standardized patient (SP) role-play remains a popular training method in which a trained actor assumes the role of a patient, engages in a simulated conversation with a clinician, and provides actionable feedback. Live SP exercises are limited by cost, availability, skill, demographic diversity, and consistency [17-20]. As a result, many clinicians lack access to high-quality, repeated practice, leaving them underprepared. Scalable, easily accessible alternatives are needed to support widespread development of communication skills.

To fill this gap, AI-driven virtual patients offer a promising solution. Prior work has explored conversational agents for providing psychotherapy [21], communication skills training [22], AI-assisted feedback [23], and empathetic response generation [24]. Recent studies have demonstrated the feasibility, usability, and scalability of large language model (LLM)–based virtual patient systems for medical education, including history-taking and simulated clinician-patient dialogue with feedback, primarily through usability studies, systematic reviews, intrinsic evaluations, and trials relying on self-reported or system-based outcomes rather than blinded behavior assessment [25-30]. Despite these promising developments, it is necessary to determine whether AI systems can help cultivate communication skills that clinicians can easily apply in practice. To our knowledge, no published randomized controlled trial (RCT) has evaluated a generative AI–based virtual patient system with personalized, structured feedback and multirater-validated behavior outcomes.

We developed SOPHIE (Standardized Online Patient for Healthcare Interaction Education), a generative AI–powered virtual patient designed to support SIC training. SOPHIE simulates conversations with a patient with advanced cancer and provides real-time, structured feedback on 3 core communication skills—Empathize, Be Explicit, and Empower (3Es)—grounded in the MVP (medical situation, values, and plan) communication framework [31]. Drawing from previous work on clinical communication [32-34] and principles of deliberate practice and behavior learning [35-37], SOPHIE enables repeated, psychologically safe practice through interactive dialogue and automated feedback. Building upon our previous work in schema and in an LLM-based virtual patient system, this work introduces a hybrid dialogue manager that is controllable and versatile. The system was iteratively refined through qualitative clinical evaluation [38] and a controlled pilot study assessing feasibility [39].

In this study, we conducted an RCT to evaluate whether training with SOPHIE leads to greater improvement in SIC skills and self-reported confidence among health professionals compared with written educational materials. We hypothesized that participants assigned to SOPHIE would show greater pre-to-post improvement in Empathize, Be Explicit, Empower, and overall communication scores than participants assigned to the control condition.

## Methods

### Study Design

We conducted a single-blind, parallel-group, RCT to evaluate whether an AI-powered virtual patient (SOPHIE) could improve clinician communication skills in serious illness conversations. Participants were randomized in a 1:1 ratio to the intervention (SOPHIE training) or the control (reading modules) group. The study is reported in accordance with the CONSORT 2025 Statement for randomized trials, and the completed CONSORT (Consolidated Standards of Reporting Trials) checklist is provided in Checklist 1.

### Sample Size Calculation

We performed a power analysis using pilot data that showed an effect size of 0.82 (Cohen *d*) to determine the target sample size for the study. A 1-tailed, 2-sample *t* test at a 5% type I error rate was selected to detect improvement in the SOPHIE arm relative to the control. We determined that a minimum of 48 participants (24 per arm) was needed to achieve at least 80% power. Sessions were recorded for blinded evaluation. Control participants were offered SOPHIE training after the study. Figure 1 demonstrates the RCT study design.

**Figure 1.**
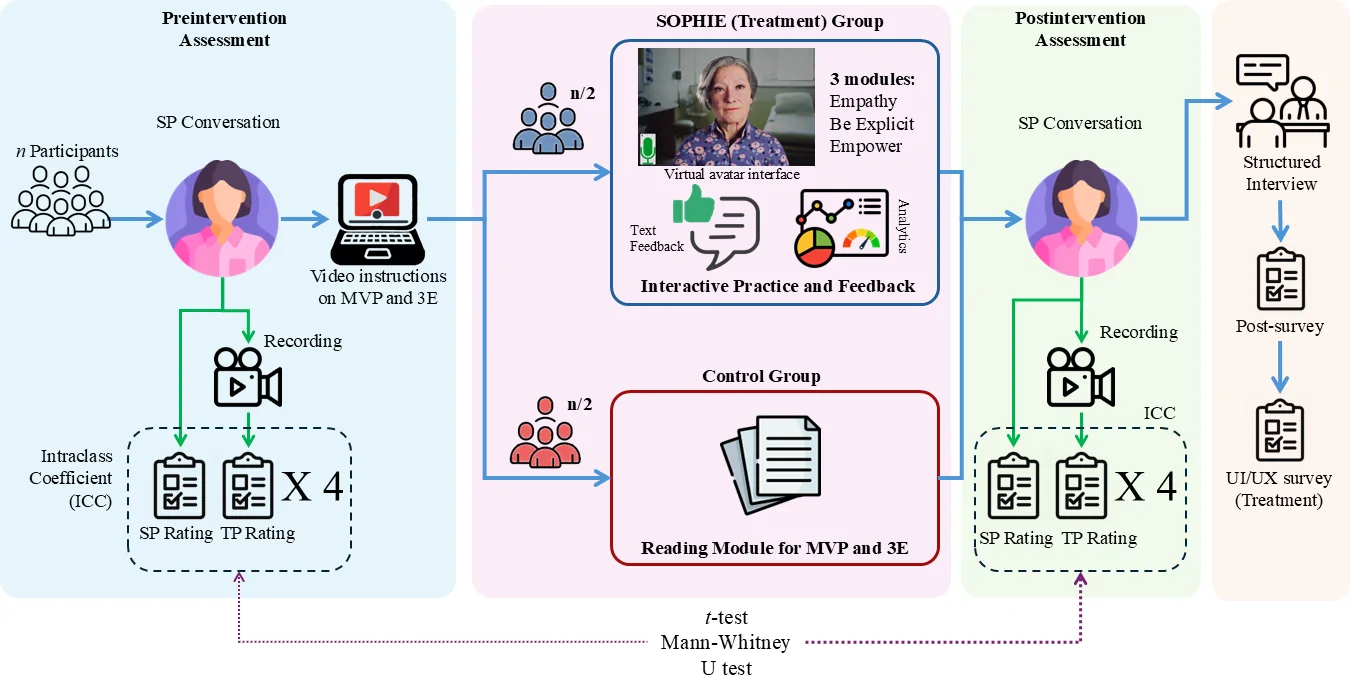
Randomized controlled study design. Participants were assigned to either the control group (reading module) or the SOPHIE (Standardized Online Patient for Healthcare Interaction Education) group (AI-guided simulation). All participants completed standardized patient (SP) conversations before and after the intervention, which were scored by the SP and 4 third-party (TP) raters. MVP (medical situation, values, and plan) and 3Es (Empathize, Be Explicit, and Empower) [31] are a communication protocol and skills, respectively. UI: user interface; UX: user experience.

### Recruitment and Randomization

We enrolled 51 participants, including medical, nursing, and physician assistant students, as well as practicing clinicians, from June to December 2024. Most participants were recruited from the University of Rochester School of Medicine and Dentistry, the University of Rochester School of Nursing, and Strong Memorial Hospital. Recruitment was conducted via email outreach, personal contacts, and campus flyers. Participants provided informed consent before participation. The study took place via the online video-calling platform Zoom (Zoom Communications Inc). Participants were randomized to the SOPHIE intervention (n=26) or the control (n=25). We used stratified block randomization to ensure balance across different participant classes (eg, trainees vs professionals). Prior participation in the SOPHIE pilot study was the only exclusion criterion. Each participant received a US $30 Amazon gift card as compensation. Table 1 presents the demographic breakdown of participants by age, gender, race or ethnicity, and clinical role.

**Table 1.** Participant demographics and dataset summary.

Characteristics	Control (N=25)	SOPHIE^a^ (N=26)	Total (N=51)
Age (y), mean (SD)	30.0 (11.2)	31.1 (15.1)	—^b^
Gender, n (%)			
Women	18 (72.0)	22 (84.6)	40 (78.4)
Men	7 (28.0)	3 (11.5)	10 (19.6)
Prefer not to say	0 (0.0)	1 (3.8)	1 (2.0)
Race or ethnicity, n (%)			
White	12 (48.0)	17 (65.4)	29 (56.9)
Asian	5 (20.0)	6 (23.1)	11 (21.6)
Black	3 (12.0)	0 (0.0)	3 (5.9)
Latino or Hispanic	3 (12.0)	1 (3.8)	4 (7.8)
Middle Eastern or other	2 (8.0)	2 (7.7)	4 (7.8)
Clinical role, n			
Health care students	14	14	28
Practitioners (physician assistants, nurses, PhDs, and residents)	11	12	23
Dataset summary statistics, n			
Video conversations	—	—	102
Reviews collected	—	—	506
Standardized patient actors	—	—	13
Third-party raters	—	—	4
Reviews per video	—	—	5
Case distribution, n			
Case I: stage IIIa lung cancer	—	—	168
Case II: metastatic lung cancer	—	—	183
Case III: renal cell carcinoma	—	—	155

a SOPHIE: Standardized Online Patient for Healthcare Interaction Education.

b Not applicable.

### Intervention and Control Conditions

All participants viewed an instructional video on SIC and how to use the MVP framework and 3E skills [31]. The intervention group completed 3 SOPHIE modules, each focusing on 1 of 3 communication skills—Empathize, Be Explicit, and Empower—combining real-time, interactive dialogue with a virtual patient and immediate automated feedback. The Empathize module focused on responding to emotional distress, the Be Explicit module on clearly communicating prognosis and uncertainty, and the Empower module on eliciting values and supporting a patient-centered care plan. After each module, participants received personalized feedback on effective behaviors, missed opportunities, and suggested alternatives for the specific module. Examples of feedback and interface visuals are shown in Figure 2. Figure 3 shows a participant navigating through one of the SOPHIE modules and pre-SP and post-SP interactions.

Control participants reviewed reading materials on the same SIC framework for the same equal 30-minute duration. The reading materials used in the control arm covered the same SIC framework and the same 3 target skills addressed in the SOPHIE modules: Empathize, Be Explicit, and Empower. Thus, both groups received the same core educational content, but only the SOPHIE group had the opportunity to practice these skills interactively with a virtual patient and to receive automated, personalized feedback. Control participants reviewed the reading materials for the same 30-minute duration as the SOPHIE training to maintain comparable exposure time across arms.

**Figure 2.**
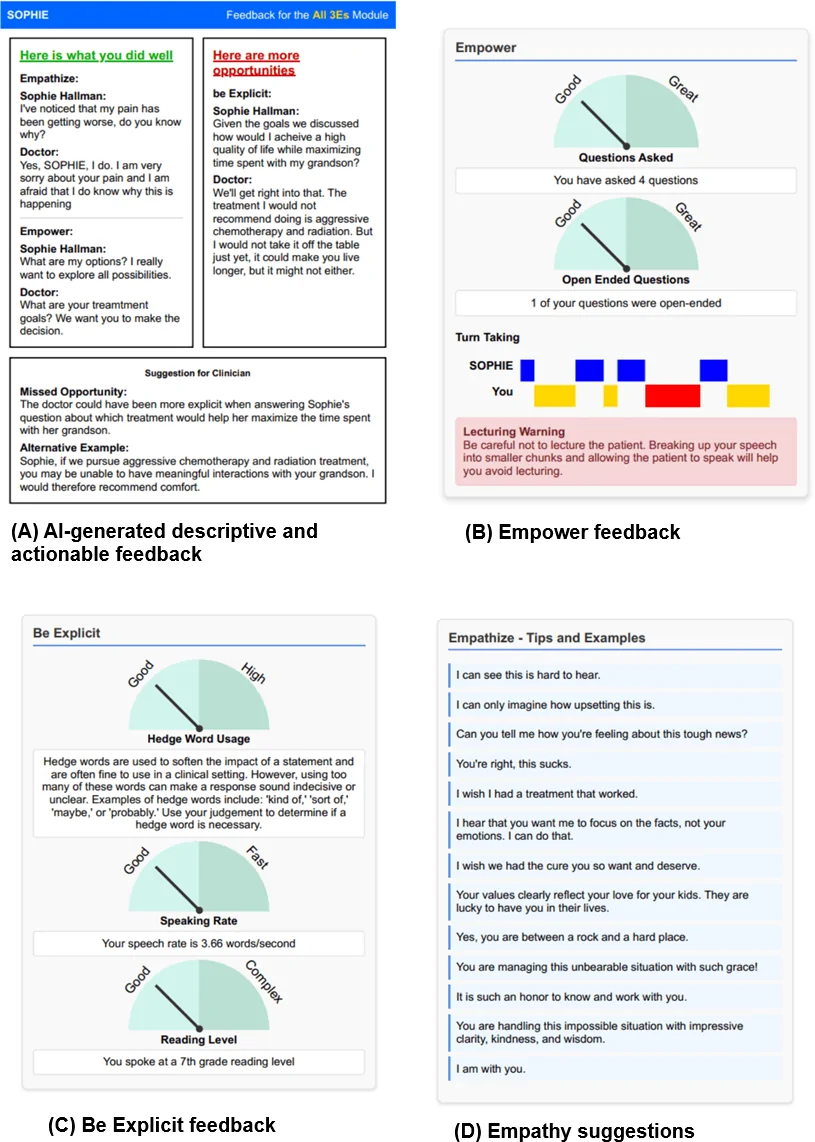
SOPHIE (Standardized Online Patient for Healthcare Interaction Education) feedback interface, simplified for visualization. Panel A shows the main dashboard with skill-based feedback. Panels B to D provide targeted guidance for the Empower, Be Explicit, and Empathize domains.

**Figure 3.**
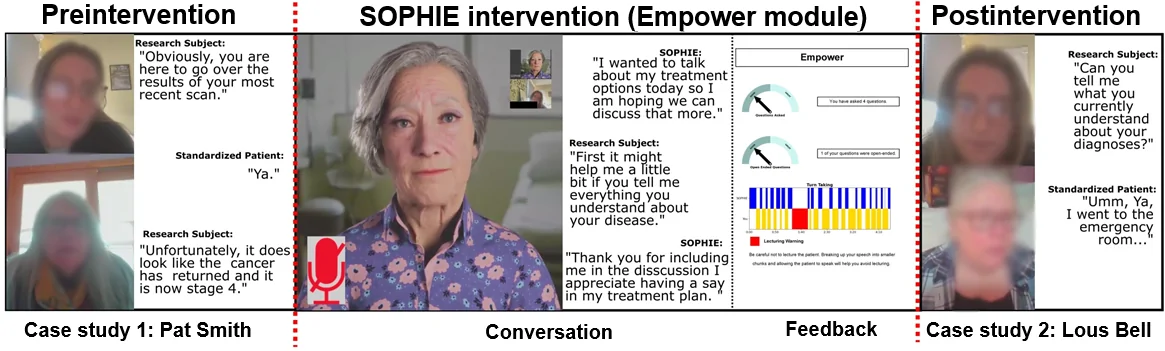
Illustration of the impact of SOPHIE’s (Standardized Online Patient for Healthcare Interaction Education) Empower module on communication. A participant initially delivers difficult news with limited patient engagement. Following SOPHIE’s training and targeted feedback, the participant adopts a patient-centered approach, using open-ended questions to foster understanding and shared decision-making.

### SP Encounters

All participants completed preintervention and postintervention simulated encounters with a trained SP drawn from 1 of 3 patient scenarios. Each participant was paired with 1 of 13 SPs, who interacted with the participant via video conference and rated the participant’s communication skills using the previously described evaluation rubric [31,39]. The 13 SPs who directly interacted with participants were hired through an external service (Professional Communication Simulators). In each interaction, SPs were randomly assigned to 1 of 3 advanced cancer clinical cases (Table 1, case distribution) developed in collaboration with experts in communication training (RME and TC). The SPs were blinded to group assignment (intervention or control) but were aware of the encounter order (pre or post). We recorded a video of each participant-SP encounter and 4 additional actors, who work with the ACT Program (Advanced Communication Training Program, University of Rochester), which is directed by one of the authors (TC), watched the videos and provided a separate rating for both preintervention and postintervention participant-SP encounters. These actors were blinded to both the intervention and the encounter order. Both SPs and third-party (TP) raters completed an online form, described in Table S2 of Multimedia Appendix 1, to evaluate the participant.

### Postinteraction Survey and Interview

After the intervention, all participants completed a postinteraction survey and a 10-minute interview (Tables S3-S7 in Multimedia Appendix 1). The shared survey asked both control and SOPHIE participants to rate perceived improvements in Empathize, Be Explicit, Empower, and overall communication skills, as well as stress during the SP interactions. Arm-specific survey questions differed by condition: control participants evaluated the usefulness and engagement of the reading materials, whereas SOPHIE participants evaluated the usefulness, engagement, stress, personalized feedback, and willingness to repeat the SOPHIE modules.

The interview included both structured and open-ended questions. Structured prompts asked all participants what they learned, what messages they found most important, whether the training improved their confidence or competence in the 3E skills, and whether they preferred interactive systems, passive materials, or a combination for communication training. Open-ended prompts allowed participants to describe their emotional experience, expected clinical takeaways, concerns, and suggestions for an ideal computer-based training system. We conducted and recorded the interviews through Zoom immediately after the experiment. These recordings were reviewed, and major themes were extracted from participant responses.

### System Design

#### Overview

The SOPHIE system features a high-fidelity, lifelike virtual patient—a 60-year-old woman with incurable stage 4 lung cancer. Users engage with the system via a face-to-face video call and, after the interaction, receive personalized feedback based on the conversation. Together, the interactive virtual patient encounter and the automated feedback system constitute the SOPHIE intervention. Multimedia Appendix 2 provides a computational cost breakdown for operating our conversation system.

#### Virtual Avatar Interface

The virtual avatar used in this study was created using the Unreal Engine MetaHuman platform, a 3D computer graphics game engine developed by Epic Games. When the user speaks, their speech is transcribed by a speech-to-text model and processed by the dialogue manager. The dialogue manager generates a response and a context-appropriate emotion tag. The response is converted to audio using a text-to-speech system. For both speech recognition and synthesis, we use a commercial API, the Microsoft Azure Speech SDK. Using the emotion tag, the avatar can express emotions through facial expressions and voice. Alongside a neutral expression, the avatar can express 6 basic emotions through facial expressions: happy, sad, angry, disgusted, surprised, and afraid. Figure 4 displays examples of these emotional facial expressions. The facial expressions are prerecorded motion-capture animations created by the authors; when triggered, the necessary animation file is read from storage and streamed to the avatar. Figure 5 shows the system’s data flow diagram.

**Figure 4.**
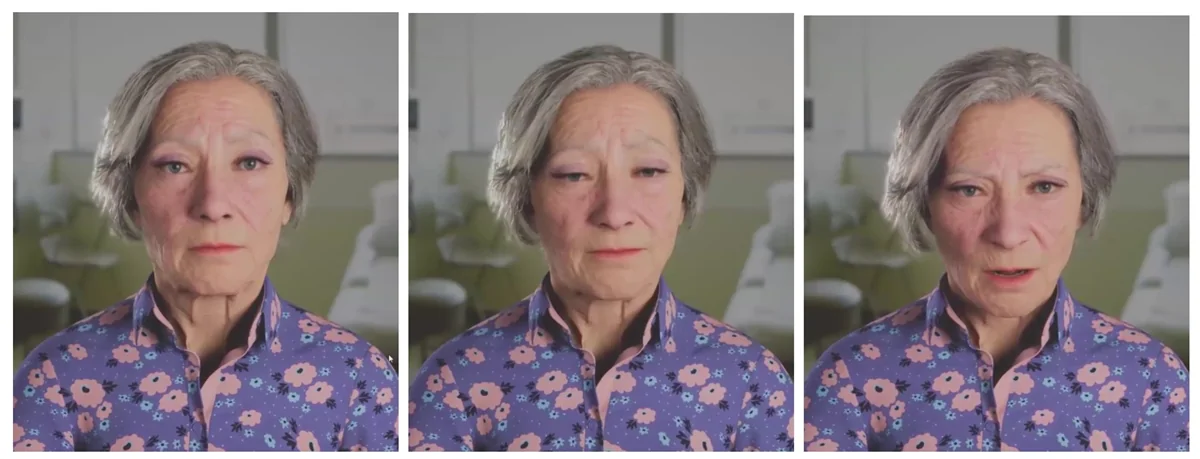
SOPHIE (Standardized Online Patient for Healthcare Interaction Education) avatar emotional expressions: neutral (left), sad (middle), angry (right). Additional emotions include happy, surprise, fear, and disgust.

**Figure 5.**
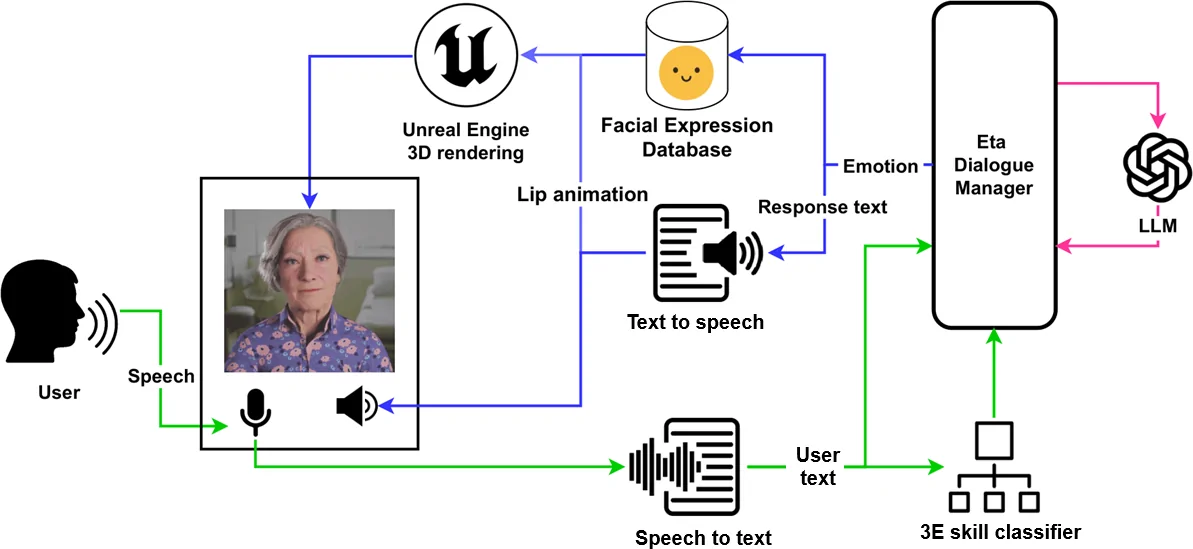
SOPHIE (Standardized Online Patient for Healthcare Interaction Education) user speech-to-avatar response generation flow. The user’s speech is transcribed using a speech-to-text model, after which a dialogue manager guided by a large language model (LLM) decides the response and emotion. Then the response is played as audio using a text-to-speech model, and facial animation and emotion are displayed with the 3D avatar in Unreal Engine. The pipeline executes in near–real time. 3E: Empathize, Be Explicit, and Empower.

#### Dialogue Manager: Schema-Guided+LLM Hybrid

SOPHIE uses a hybrid dialogue system that balances pedagogical control with naturalistic interaction. In our previous work, we have explored using solely a schema-based dialogue system [34,39] and a fully LLM-based dialogue system [40,41], both of which had important limitations. The schema-based dialogue system is more controllable; however, the responses lack diversity and feel scripted. On the other hand, an LLM-based dialogue system, although more natural, is prone to diverging from the topic. In particular, older models such as ChatGPT (GPT-3.5 Turbo; OpenAI) were highly prone to hallucinating and drifting from the main conversation topic.

To create a controllable yet natural conversation system, we developed a hybrid dialog manager that uses a combination of a schema-based system and an LLM. The schema system is designed with the help of actual SIC conversations from the dataset in Epstein et al [33] and grounds the dialogue and provides guardrails. The schema also encodes the expected flow and communication goals per module (Empathize, Be Explicit, and Empower), tracks conversation state, and detects relevant user actions. Figure 6 demonstrates how such a skill is classified based on a user’s utterance using a combination of rule-based and machine language–based classifiers. The schema system used in this study is a variation of the dialogue system described in Kane et al [34]. However, the main difference is that when the user’s input aligns with one of the predefined schemas, instead of generating a full response, the system generates a structured template for SOPHIE’s responses, including an emotion tag representing how the virtual patient is feeling. Next, an LLM (GPT-3.5 Turbo) is instructed to respond naturally to the user, strictly following the template. If the user’s input does not match any predefined schema patterns, a response is generated directly by the LLM. This hybrid method gives SOPHIE the controllability and guardrails of schema-based dialogue, while leveraging the versatility of LLMs. The schema and structured prompting components are intended to reduce dependence on the underlying LLM, although robustness across different models has not yet been evaluated.

**Figure 6.**
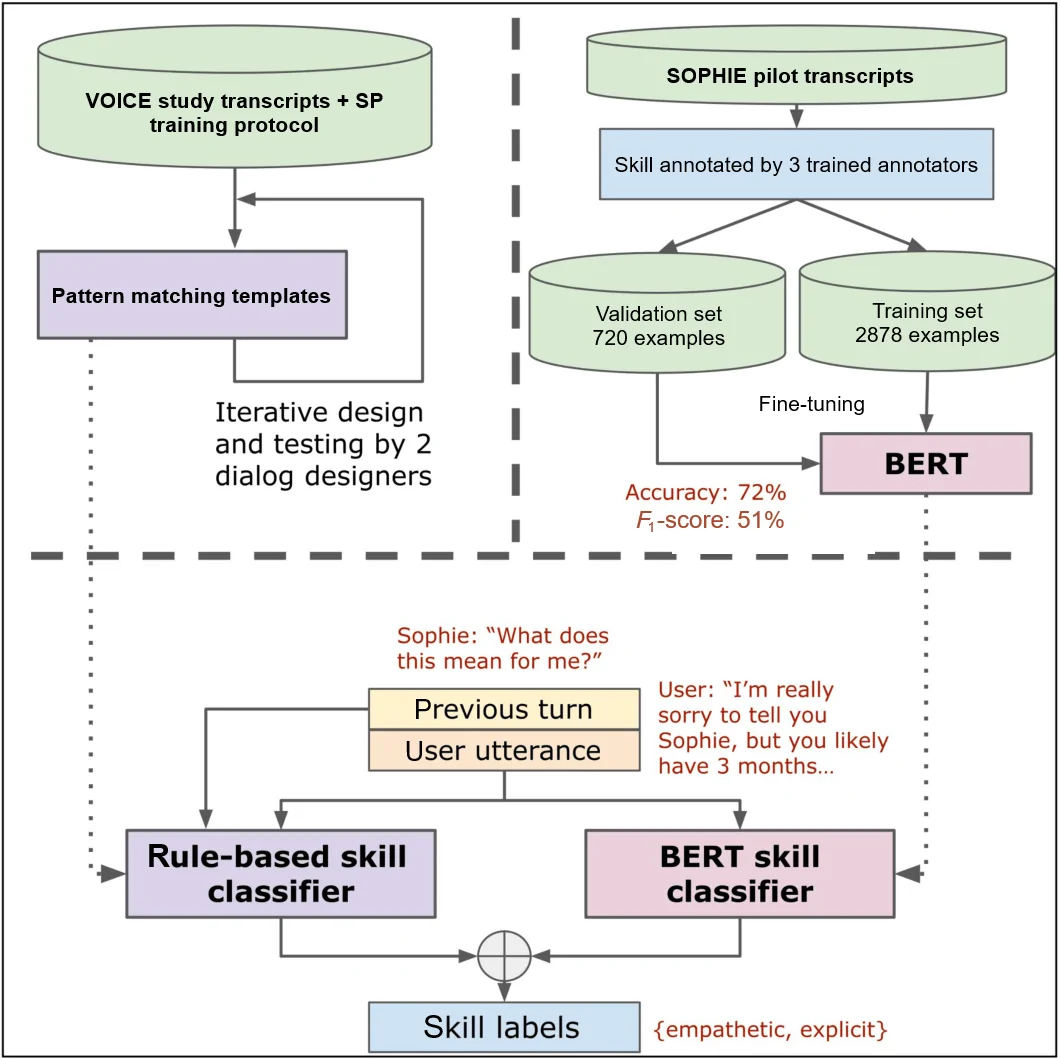
A hybrid skill classifier that detects whether a statement from the user falls within the 3E (Empathize, Be Explicit, and Empower) skills. BERT (Bidirectional Encoder Representations from Transformers) is a pretrained deep learning model for text classification [42], and VOICE (Values and Options in Cancer Care) is a dataset consisting of real cancer care conversation transcripts [33]. SOPHIE: Standardized Online Patient for Healthcare Interaction Education; SP: standardized patient.

#### Feedback Delivery

The feedback model is grounded in deliberate practice and behavioral learning theory [35-37], co-designed with practicing clinicians and refined through pilot testing [38,39]. Following each SOPHIE module, users receive structured feedback aligned with the 3 target skills: Empathize, Be Explicit, and Empower. The feedback includes a combination of personalized LLM-generated feedback, examples of what the participant did well and of missed opportunities, suggested alternative phrasing, and skill-specific, structured metrics. The LLM-generated feedback is grounded in the MVP framework and is thus robust to LLM changes. In structured feedback, the Empower module provides feedback on questions asked, open-ended questions, and turn-taking; the Be Explicit module provides feedback on hedge words, speaking rate, and reading level; and the Empathize module provides example phrases for responding to patient emotion. All these metrics were carefully designed in previous studies to elicit each clinical communication skill [38,39]. Figure 2 shows examples of the feedback interface. Figure 7 shows an example of line-by-line feedback on the participant’s conversation.

**Figure 7.**
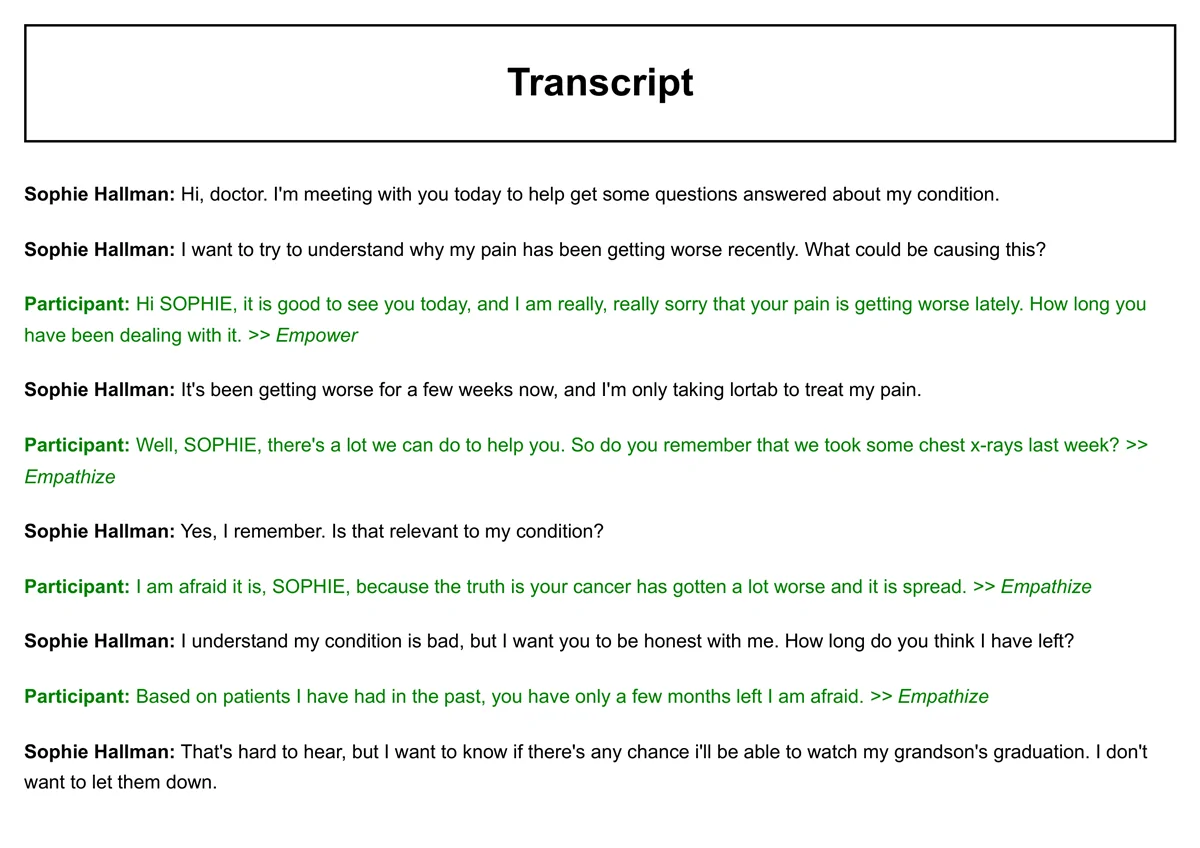
Part of a sample transcript from detailed feedback with the 3E (Empathize, Be Explicit, and Empower) skill classification. SOPHIE: Standardized Online Patient for Healthcare Interaction Education.

### Scoring Rubrics to Measure Communication Skills

To our knowledge, there are no widely established benchmark scores for SIC performance. We therefore adopted the MVP framework and converted the 3 target skills—Empathize, Be Explicit, and Empower—into a structured Likert-scale questionnaire to quantify communication behaviors on a numeric scale. The full set of statements used in the rubric is provided in Table S2 of Multimedia Appendix 1. For each of the 3E skills (*S*), we summed the Likert scores associated with the skill and normalized them to between 0 and 1 using equation 1. For the overall score, questions related to all 3 skills were summed. This process was repeated for the ratings provided by the SP and the 4 TPs, and the resulting scores were averaged:


(1) $${Score}_{S}=\frac{1}{5}\sum_{i=SP,\;{TP}_{1-4}}\left(\frac{\sum_{j\in \{{question\_set}_{S}\}}{Likert\_score\_value}_{j,i}}{max\_point\_{possible}_{S}}\right).$$

We averaged the evaluations across 5 raters to reduce the effects of individual biases and normalized the scores to between 0 and 1 to make them interpretable and comparable across raters.

The rubric was intended to capture within-participant improvement from before to after the intervention, not to define an ideal level of performance or estimate population-level clinical competence. Future studies may use a similar rubric to evaluate whether repeated SOPHIE practice produces larger or more durable gains over time.

### Statistical Procedures

We summarized participant demographics using descriptive statistics. Communication skill outcomes were analyzed for each of the 3Es (Empathize, Be Explicit, and Empower) and for the overall score. For descriptive reporting, we calculated pre-to-post change scores by subtracting each participant’s first SP encounter score from their second encounter score.

To test whether improvement differed by study arm while accounting for repeated measurements within participants, we fit separate linear mixed effects models for Empower, Be Explicit, Empathize, and overall communication score. Each model included fixed effects for study arm, time (first vs second SP encounter), and the study arm-by-time interaction, with a random intercept for each participant to account for within-participant correlation. Models were estimated using maximum likelihood under an intention-to-treat framework, including all randomized participants, and were specified as:

score *~* study arm *×* time *+* (1 | participant).

The study arm-by-time interaction was the primary test of the intervention effect because it estimates the additional pre-to-post improvement in the SOPHIE arm relative to the control arm. Statistical significance was evaluated using α of .05, and 95% CIs were reported for model estimates.

### Ethical Considerations

This study was approved by the University of Rochester Institutional Review Board (IRB; study ID: STUDY00006689). All participants provided informed consent after receiving an explanation of the study procedures, risks, and benefits, and their participation was voluntary. The study was conducted remotely using Zoom, and video meetings were recorded only with participants’ consent. Study data and recordings were handled according to IRB-approved confidentiality procedures, stored in secure cloud storage, and deidentified before analysis or data sharing. Only aggregate demographic information is reported.

## Results

### Participant Characteristics

Both participant groups were demographically balanced, with no notable differences in age, gender, or clinical background (Table 1). Logistic regression confirmed no demographic predictors of group assignment. The CONSORT diagram is displayed in Figure 8. Of the 65 eligible participants who signed up, 14 did not show up at the scheduled meeting time. Of the 51 participants who showed up and signed the consent form, all completed the full study. Because the full study took place in a single, continuous session, we achieved a zero-dropout rate.

**Figure 8.**
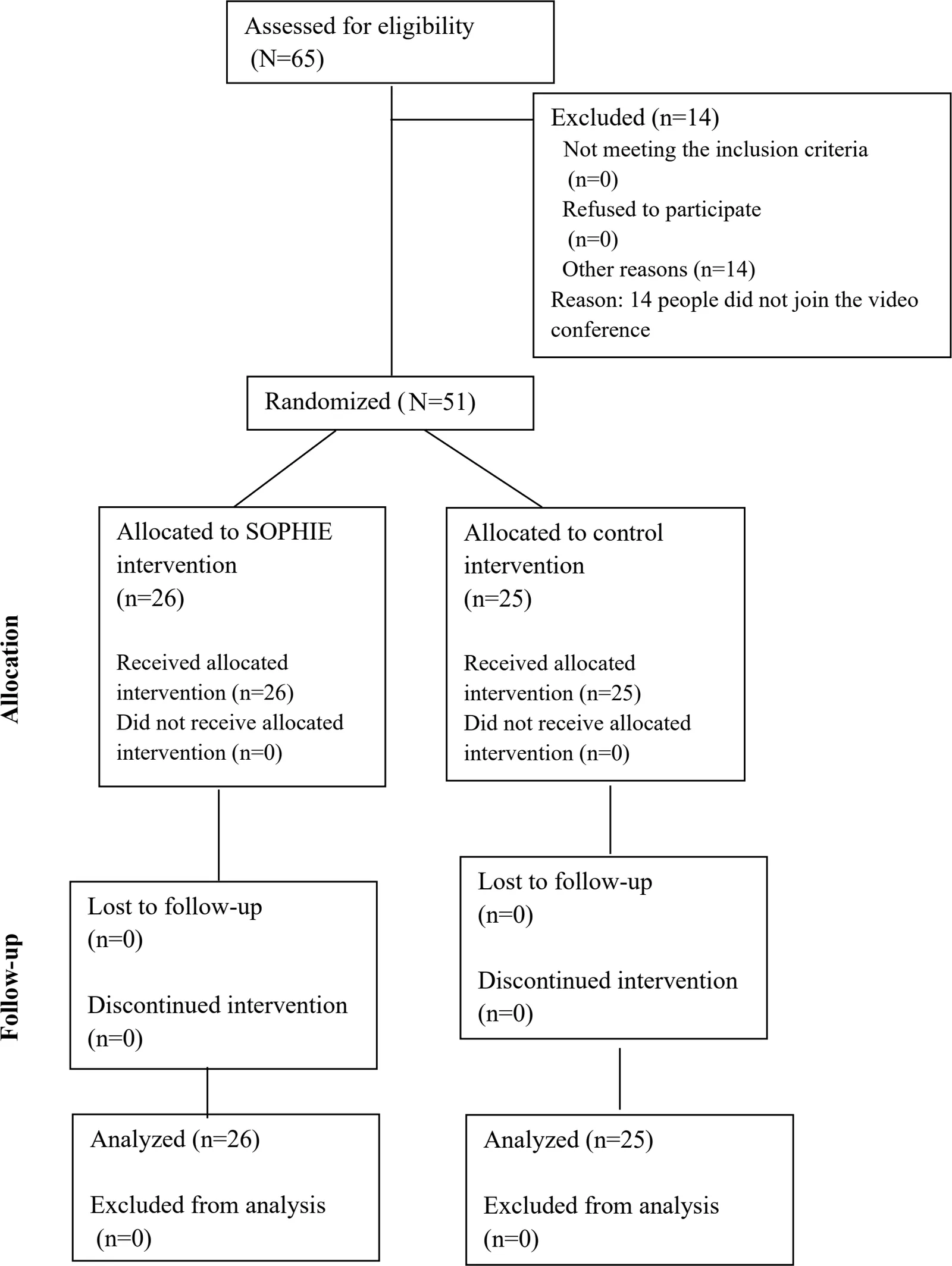
CONSORT (Consolidated Standards of Reporting Trials) diagram. SOPHIE: Standardized Online Patient for Healthcare Interaction Education.

### Interrater Agreement

We examined agreement between the SP ratings and the blinded TP rating using a leave-one-out comparison. The full analysis is reported in Table S1 of Multimedia Appendix 1. For each rater, we compared that rater’s score with a reference consensus formed by averaging the remaining raters’ scores. SP refers to the SPs who interacted directly with participants, while TP0 to TP3 refer to the 4 SPs who reviewed the recorded encounters as blinded TP raters. All ratings were normalized at the rater level before analysis to reduce individual scale-use differences.

The SP ratings showed moderate-to-strong agreement with the blinded TP rater consensus (Pearson correlation coefficient *r*=0.68; Spearman rank correlation ρ=0.67; mean absolute error=0.15; intraclass correlation coefficient [ICC] (A,1)=0.63; 95% CI 0.50‐0.74). Agreement varied across individual TP raters but was generally within a similar range, with ICC(A,1) values from 0.57 to 0.78. These results suggest that SPs and blinded TP raters captured broadly consistent communication quality while also reflecting meaningful differences in perspective between first-person interaction and TP observation.

### Primary Outcome: Communication Skill Improvement

To evaluate communication skill development, we analyzed changes in participants’ scores (Empower, Be Explicit, Empathize, and overall) between their first and second SP conversations. Each conversation (n=102) received 5 independent ratings—1 from the participating SP and 4 from blinded TP reviewers. Final scores were averaged across these ratings and change scores (∆) were calculated by subtracting preintervention from postintervention values and then aggregated as described earlier. Of the expected 510 ratings from 51 participants across 2 SP encounters and 4 independent raters, 4 ratings were missing, resulting in 506 ratings for the final analysis. Interrater reliability was strong, with an ICC of 0.88 (95% CI 0.82‐0.93) among TP reviewers blinded to intervention or control status before and after the study.

Both groups showed improvement from the first to the second SP encounter, but the SOPHIE group showed larger gains across all communication outcomes (Table 2). For Empower, mean scores increased from 0.49 to 0.55 (Δ=0.06) in the control group and from 0.41 to 0.58 (Δ=0.17) in the SOPHIE group. Similar patterns were observed for Be Explicit (control Δ=0.05; SOPHIE Δ=0.13), Empathize (control Δ=0.07; SOPHIE Δ=0.14), and overall score (control Δ=0.06; SOPHIE Δ=0.15).

To account for the repeated-measures structure of the randomized pre-post design, we analyzed communication outcomes using linear mixed effects models under an intention-to-treat framework. All randomized participants (n=51; 102 observations) were included in the analyses.

The mixed effects models showed significant study arm-by-time interactions for all outcomes. The SOPHIE arm showed greater pre-to-post improvement than the control arm for Empower (β=.11, 95% CI .04-.17; *P*=.002), Be Explicit (β=.08, 95% CI .03-.13; *P*=.002), Empathize (β=.07, 95% CI .01-.13; *P*=.03), and overall communication score (β=.09, 95% CI .04-.14; *P*=.001). In practical terms, these interaction estimates represent the additional improvement associated with SOPHIE beyond the improvement observed in the control arm.

**Table 2.** Communication skill improvement in control and SOPHIE (Standardized Online Patient for Healthcare Interaction Education) arms.

Arm	Control			SOPHIE					*P* value^a^
	Pre	Post	Δ^b^	Pre	Post	Δ^b^	LLM^c^ interaction β^d^ (95% CI^e^)		
Empower	0.49	0.55	0.06	0.41	0.58	0.17	.11 (.04-.17)		.002
Be Explicit	0.71	0.75	0.05	0.66	0.79	0.13	.08 (.03-.13)		.002
Empathize	0.58	0.65	0.07	0.51	0.65	0.14	.07 (.01-.13)		.03
Overall	0.59	0.65	0.06	0.52	0.67	0.15	.09 (.04-.14)		.001

a *P* indicates whether the difference in improvement between arms was statistically significant.

b Δ represents the difference between the second and first encounter mean scores according to the scoring rubric.

c LLM: large language model.

d The LLM interaction β value represents the additional pre-to-post improvement in the SOPHIE arm relative to the control arm, estimated from linear mixed effects models.

e The 95% CI indicates the uncertainty around this estimate.

### Secondary Outcomes: Learner Perceptions and System Efficacy

Participants using the SOPHIE system reported markedly greater confidence in their communication skill development compared with controls on a Likert scale ranging from 1 to 5, with average perceived improvement scores of 4.31 (SOPHIE) vs 3.83 (control) on a 5-point scale (*P*=.009).

The training system itself received strong evaluations, particularly for its actionable feedback on communication skills—the highest-rated feature (mean: 4.7/5, SD: 0.18). Users also praised SOPHIE’s usability (mean: 4.2/5, SD: 0.36), though ratings for the virtual human’s realism were more moderate (mean: 3.4/5, SD: 0.17).

### Qualitative Analysis

#### Overview

Qualitative feedback from post-session participant interviews was video-recorded and analyzed by the author KGH, revealing 3 dominant themes: the inherent difficulty of serious illness conversations, SOPHIE’s value as a training tool, and opportunities for system refinement.

#### Apprehension in SIC

Participants universally acknowledged the emotional complexity of serious illness dialogues, with many expressing surprise at their own discomfort. One participant admitted, “I felt nervous—even really, really bad—practicing these conversations,” while another emphasized, “Doctors are human too—nobody is born an expert.” Participants generally described this discomfort as arising from the emotionally difficult nature of SIC rather than from the SOPHIE system itself. One of the study authors (KGH) remained available throughout each interaction to provide support if needed. No participant discontinued the session or requested additional support because of distress.

#### Training Value of SOPHIE

The system’s accessibility and actionable feedback were widely praised. Participants likened the experience to “being trained in a facility by a specialist, but from my laptop,” highlighting its convenience without compromising pedagogical rigor. The specificity of feedback—described as “actionable guidance, not just generic encouragement”—enabled targeted skill development. Notably, the judgment-free environment empowered experimentation: “I tried new strategies because there was no risk of hurting a real patient,” one participant explained. Repeated practice translated to tangible confidence gains, with users reporting feeling “more prepared for real conversations.”

#### System Refinement

While effective, SOPHIE’s realism faced criticism. Participants noted “creepy” or “uncanny” interactions due to limited emotional range, abrupt speech patterns, and lack of eye contact. Robotic reinforcement phrases (eg, “Thank you for allowing me to express my emotions”) further reduced perceived authenticity. To address these gaps, users proposed enhancing multimodal analysis (facial expressions, tone, body language) and integrating game elements (eg, performance scoring and competition).

Beyond health care, participants recognized the 3E framework’s potential in business, law, education, and personal relationships.

## Discussion

### Principal Findings

This RCT tested whether SOPHIE would lead to greater improvement in SIC skills and confidence than did written educational materials. The findings support this hypothesis: participants assigned to SOPHIE showed greater pre-to-post improvement than controls across Empower, Be Explicit, Empathize, and overall communication scores, as well as higher self-reported confidence. These gains occurred after a brief training session of less than 30 minutes, suggesting that AI-based virtual patient training may be feasible within clinical education settings.

Confidence gains were also higher with SOPHIE, indicating that interactive practice not only improved competence but also helped learners recognize their progress. Interviews reinforced this: many participants felt underprepared for the emotional demands of SIC, underscoring the value of deliberate, low-risk practice.

Participants rated SOPHIE’s actionable feedback (4.7/5) higher than the avatar’s visual realism (3.4/5), suggesting that visual fidelity has room for improvement.

### Comparison With Prior Works

Prior work on LLM-based virtual patients has largely focused on system feasibility, usability, and intrinsic quality rather than on behavior learning outcomes. Studies by Elhilali et al [25] and Cook et al [27] demonstrate that LLM-driven patient simulations can generate realistic dialogue and are perceived as usable, but they do not assess whether such systems improve clinician communication skills. A recent systematic review [26] similarly highlights the lack of consistent outcome measures and the limited evidence of skill transfer in the literature. Importantly, very little published work has evaluated AI-based training specifically for SIC, and the available literature is particularly limited in randomized trials with behavior outcomes.

Some randomized studies evaluate AI-based simulations but rely mainly on self-reported outcomes. Tyrrell et al [29] and Roux et al [30] report improvements in perceived skills or confidence, yet do not show clear gains in objectively assessed communication behaviors. Other work using virtual SPs without LLM-driven dialogue has demonstrated improvements in clinical reasoning and diagnostic skills [28], but these systems do not target conversational communication or provide generative, personalized feedback. Thus, although AI-based virtual patient systems are increasingly being studied in medical education, there remains a dearth of evidence on whether such systems can improve SIC skills that transfer to human SP encounters.

In contrast, this study evaluates an LLM-based virtual patient designed for deliberate practice in SIC and measures effectiveness using a randomized controlled design with multirater behavior assessments. This work extends prior studies by providing evidence that generative AI–based patient simulations can lead to measurable improvements in clinician communication behaviors beyond usability or self-reported learning gains.

### Future Directions and Scalability

As health systems embrace telemedicine and remote education, scalable and equitable training is essential. SOPHIE leverages generative AI to standardize, measure, and democratize communication training, with the potential to mitigate bias by enabling practice with diverse patient identities. Even modest clinician gains at scale could reduce disparities in outcomes. Applications in conflict resolution, medical error disclosure, and misinformation highlight its versatility. Figure 9 demonstrates diverse appearances of virtual avatars that can be easily integrated into our system.

These findings provide emerging empirical support for the hypothesis that AI can serve as an adaptive tutor in complex interpersonal domains [35]. By enabling deliberate practice with structured, immediate feedback [36,37,43], AI systems can support skill development traditionally cultivated through in-person mentorship.

Importantly, our assessment focused not only on participant engagement with the training platform but also on whether skills translated to performance in encounters with SPs. This approach supports the evaluation of skill transfer beyond the virtual training environment—an important consideration for tools intended to support clinical practice. Looking ahead, systems like SOPHIE may serve as a foundation for the use of AI that strengthens, rather than replaces, the clinician’s voice in moments that matter most.

**Figure 9.**
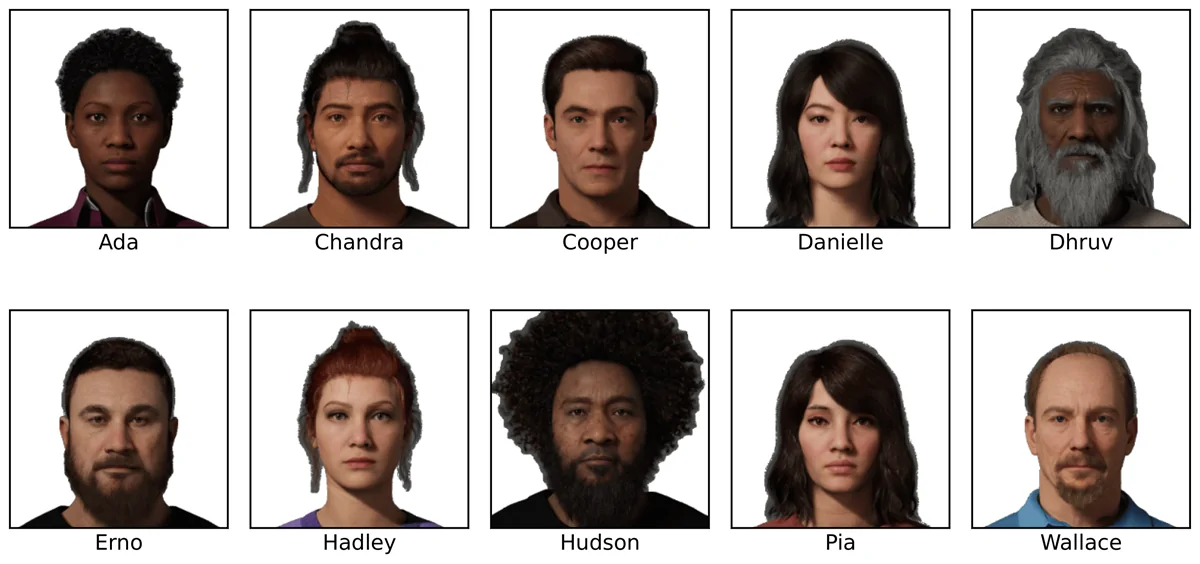
Virtual avatars can be customized to cover a wide range of demographics, including race, gender, age, and cultural background. This could potentially enable clinicians to practice highly sensitive communication in ways that were previously difficult to achieve at scale with real humans.

### Limitations

Although our approach has several strengths, the long-term effects of SOPHIE-based training remain unknown. Additionally, the assessment of improvements in communication skills was conducted in a simulated setting; future work is needed to assess its real-world clinical impact. The observed learning effects may also have been influenced by novelty or motivational effects, as many participants were likely interacting with a virtual patient for the first time. In addition, the single-scenario training, the relatively small sample size, and the same-day interaction and evaluation limit the generalizability of the findings. Our control condition measured the effectiveness of SOPHIE against a static reading condition. However, how the SOPHIE intervention would compare with other forms of interactive training, such as SP-based training and feedback, remains still unknown. Future studies should include more diverse virtual patients and clinical cases, larger samples, longer follow-up periods, and comparisons of AI training with human training. Four of the expected 510 ratings were missing; given the small proportion of missing ratings, their effect was likely limited, although some measurement uncertainty remains.

Our dialogue agent captures the user’s speech by transcribing it into text and does not take into account the user’s facial expressions, prosody, intonation, body language, or other nonverbal cues. While participants recognized emotions such as sadness and concern during interactions, some noted that SOPHIE’s expressions felt “uncanny.” This highlights a common design challenge: avatars that look too human can be unsettling, while more stylized expressions may actually make emotional cues clearer. Whether intentionally favoring exaggerated, easy-to-read expressions is educationally superior to striving for subtle, lifelike realism should be explored further in future studies.

One important limitation of the SOPHIE system is its dependence on commercial providers for LLMs, speech recognition, and speech synthesis. This reliance can hinder exact reproducibility over time because cloud-based models and APIs may be updated or deprecated. In addition, transmitting conversation data to external services may limit the use of SOPHIE for protected or highly sensitive clinical conversations, even when appropriate safeguards are in place. These concerns could be mitigated by deploying local models, such as the Llama 3 [44] or Qwen 2.5 [45] families for language generation, Whisper [46] for speech recognition, and Kokorofor speech synthesis, allowing all processing to remain within institutional infrastructure. However, such deployments introduce additional engineering complexity and require dedicated hardware resources and ongoing maintenance, potentially increasing the operational burden for academic or health care institutions.

### Conclusions

Looking ahead, systems like SOPHIE may serve as a foundation for a new kind of AI-human partnership in health care—one that strengthens, rather than replaces, the clinician’s voice in moments that matter most.

## Supplementary material

10.2196/93034 Multimedia Appendix 1 Additional tables.

10.2196/93034 Multimedia Appendix 2 Additional methods.

10.2196/93034 Checklist 1 CONSORT checklist.

## Abbreviations

3Es: Empathize, Be Explicit, and Empower
ACT Program: Advanced Communication Training Program
CONSORT: Consolidated Standards of Reporting Trials
ICC: intraclass correlation coefficient
LLM: large language model
MVP: medical situation, values, and plan
RCT: randomized controlled trial
SIC: serious illness communication
SOPHIE: Standardized Online Patient for Healthcare Interaction Education
SP: standardized patient
TP: third party
