# Supplementary material for: Effects of a Generative AI Virtual Patient on Serious Illness Communication Skills: Randomized Controlled Trial
Source: JMIR Med Educ. 2026 Oct 20;12:e93034. doi: 10.2196/93034 (PMC13683997; doi:10.2196/93034)
Supplement: Supplementary file 2 [file mededu-v12-e93034-s001.docx]

Multimedia Appendix

Table of Contents

[Additional Tables	2](#_Toc241834652)

[Table S1: Interrater agreement between raters. SP = expert standardized patients who interacted with participant directly and provided rating. TP = blind third-party expert raters who watch the participant and SP interacting and provided independent rating.	2](#_Toc241834653)

[Table S2: Evaluation questions used to assess participant communication skills across the Empower, be Explicit, and Empathize question sets. Overall questions (7,13, 18) are on a 1-10 scale, and others are on a 1-5 scale.	3](#_Toc241834654)

[Table S3: Shared Participant Survey Questions (Control and SOPHIE Arms)	4](#_Toc241834655)

[Table S4: Arm-Specific Survey Questions	5](#_Toc241834656)

[Table S5: Shared interview questions asked of both Control and SOPHIE participants. The interviews explored participant perceptions of the training experience, communication skills learned, and future application in clinical practice.	6](#_Toc241834657)

[Table S6: SOPHIE UI/UX Survey: System Interaction and Perception	7](#_Toc241834658)

[Table S7: SOPHIE UI/UX Survey: Feedback Format and Skill Modules	8](#_Toc241834659)

# Additional Tables

## Table S1: Interrater agreement between raters. SP = expert standardized patients who interacted with participant directly and provided rating. TP = blind third-party expert raters who watch the participant and SP interacting and provided independent rating.

| **Rater** | **Reference consensus** | **Pearson↑** | **Spearman↑** | **MAE↓** | **ICC(A,1)** | **95% CI** |
| --- | --- | --- | --- | --- | --- | --- |
| SP | TP0, TP1, TP2, TP3 | 0.68 | 0.67 | 0.16 | 0.63 | [0.49, 0.74] |
| TP0 | SP, TP1, TP2, TP3 | 0.80 | 0.80 | 0.11 | 0.78 | [0.68, 0.85] |
| TP1 | SP, TP0, TP2, TP3 | 0.63 | 0.56 | 0.15 | 0.60 | [0.43, 0.72] |
| TP2 | SP, TP0, TP1, TP3 | 0.83 | 0.81 | 0.13 | 0.74 | [0.62, 0.83] |
| TP3 | SP, TP0, TP1, TP2 | 0.64 | 0.58 | 0.13 | 0.64 | [0.50, 0.74] |

## Table S2: Evaluation questions used to assess participant communication skills across the Empower, be Explicit, and Empathize question sets. Overall questions (7,13, 18) are on a 1-10 scale, and others are on a 1-5 scale.

|  | **Communication Skill Evaluation Questions** |
| --- | --- |
| **Empower Questions** | |
| 1 | The participant elicited the patient’s major concerns within the first 5 minutes of the conversation. |
| 2 | The participant asked for permission to share information about prognosis. |
| 3 | The participant asked how much information the patient would like concerning prognosis. |
| 4 | The participant checked the patient’s prognostic understanding by asking them to state what they understood, using a teach-back approach. |
| 5 | The participant actively encouraged the patient to ask questions using facilitative questions/statements (e.g., “What questions do you have?” or “At this point, many patients have questions…”). |
| 6 | The participant gave the SP multiple (2 or 3 or more) opportunities to talk. |
| 7 | Overall, I thought the participant was empowering. |
| **Be Explicit Questions** | |
| 8 | The participant described the medical situation (the cancer has spread) clearly and without euphemism or jargon. |
| 9 | The participant shared the prognosis accurately (a few weeks, a few months – less than one year). |
| 10 | The participant used clear language without euphemism or jargon when sharing the prognosis. |
| 11 | The participant used difficult-to-understand medical jargon. *(Inverted during analysis)* |
| 12 | The participant did a good job **NOT** lecturing the patient (uninterrupted information for what seemed like a long time). |
| 13 | Overall, I thought the participant was being explicit. |
| **Empathize Questions** | |
| 14 | The participant was generally empathetic. |
| 15 | The participant’s language was empathetic. |
| 16 | The participant used silence appropriately in response to patient emotion. |
| 17 | The participant validated the SP’s emotional responses. |
| 18 | Overall, I thought the participant aligned with the patient’s emotional state. |

## Table S3: Shared Participant Survey Questions (Control and SOPHIE Arms)

**Perceived Improvement of Communication Skills**

Please rate the following statements (1–5) with 5 representing strong agreement:

1. I felt genuine empathy for the standardized patient acting out the case study even though I am fully aware that the circumstances are not real.

2. Knowing that the patient and circumstances are made up makes it hard to feel real empathy.

3. Being unable to feel real empathy for the patient makes it much harder to express empathy to the patient.

4. Regardless of feeling real empathy, I believe that the appearance of being empathetic can be taught and learned.

5. I felt like my “Empathize” communication skills improved between my first and second interaction with the Human Standardized Patient.

6. Overall, I would give myself a (1–5) rating on “Empathize.”

7. I felt like my “be Explicit” communication skills improved between my first and second interaction with the Human Standardized patient.

8. Overall, I would give myself a (1–5) rating on “be Explicit.”

9. I felt like my “Empower” communication skills improved between my first and second interaction with the Human Standardized patient.

10. Overall, I would give myself a (1–5) rating on “Empower.”

11. I felt like my “Overall” communication skills improved between my first and second interaction with the Human Standardized Patient.

12. Overall, I would give myself a (1–5) rating on “Overall Communication Skills.”

13. How likely would you do the procedure again to improve or refresh communication skills?

**Stress-Related Questions**

Please rate the following statements (1–5) with 5 representing strong agreement:

1. I was stressed during my interactions with the human standardized patients.

2. It is easier for me to learn when I am relaxed and not experiencing stress.

## Table S4: Arm-Specific Survey Questions

**Control Arm**

1. I felt engaged during my reading of the paper.

2. I felt that reading the paper was useful for learning communication skills.

3. I anticipate using skills learned from doing the control procedure in clinical scenarios.

4. I would be willing to read the SAME paper again in the future to improve or refresh my communication skills.

5. I believe I would improve with this repeated re-reading.

**SOPHIE Arm**

1. I felt engaged during the SOPHIE modules.

2. I felt that the SOPHIE modules were useful for learning communication skills.

3. I anticipate using skills learned from the SOPHIE modules in clinical scenarios.

4. I would be willing to engage with the SAME SOPHIE modules again in the future to improve or refresh my communication skills.

5. I would be willing to engage with DIFFERENT SOPHIE modules again in the future to improve or refresh my communication skills.

6. Receiving incentives (e.g., CME credits) would significantly increase my willingness to use SOPHIE.

7. I was stressed while completing the SOPHIE modules.

8. Interacting with an AI is less stressful than interacting with a real human.

9. Getting personalized feedback on communication skill performance from a computer would help me improve.

10. Having the ability to practice communication skills is essential for improving them.

## Table S5: Shared interview questions asked of both Control and SOPHIE participants. The interviews explored participant perceptions of the training experience, communication skills learned, and future application in clinical practice.

**Shared Interview Questions**

1. What did you learn from participating in the experiment?

2. What do you think were the most important messages?

3. How did the program you did make you feel?

4. What did you like most about doing the program?

5. What do you think you will take away from the training into your future clinical practices?

6. Please tell me everything you can recall about the three E’s.

7. Did your training (reading a paper vs SOPHIE) help you feel more competent and/or confident Empathizing, being Explicit and Empowering? Why?

8. If you were to be a user of a computer program designed to train communication skills, what would that experience look like?

9. Would you prefer an interactive system, passive viewing materials, or a combination for end-of-life communication skills training? Why?

10. Any questions, concerns, or additional thoughts you would like to share with the research team?

## Table S6: SOPHIE UI/UX Survey: System Interaction and Perception

**System Usability**

1. I found SOPHIE system easy to use.

2. I think that I would like to use this system frequently.

3. I do NOT think that I would need the support of a technical person to be able to use this system.

4. I did NOT need to learn a lot of things before I could get going with this system.

**Avatar and Emotion Perception**

1. SOPHIE’s voice sounded human-like.

2. SOPHIE's voice showed emotion.

3. SOPHIE's voice showed different emotions.

4. SOPHIE's voice expressed sadness at times.

5. SOPHIE's voice expressed fear or concern at times.

6. SOPHIE's voice expressed frustration at times.

7. SOPHIE looked realistic.

8. SOPHIE's facial expressions expressed emotion.

9. SOPHIE's facial expressions showed different emotions.

10. SOPHIE's facial expressions expressed sadness at times.

11. SOPHIE's facial expressions expressed concern at times.

12. SOPHIE's facial expressions expressed frustration at times.

13. SOPHIE, overall, seemed to be able to express emotions through face and voice.

14. SOPHIE expressing emotion through face and voice simultaneously made the emotion easier to recognize.

15. SOPHIE expressing emotion through face and voice simultaneously made the emotion more realistic and human-like.

**Dialogue**

1. I found SOPHIE’s responses to be fluent and natural.

2. SOPHIE’s questions/comments were relevant to her medical condition.

3. SOPHIE’s responses followed logically from the conversation.

4. SOPHIE reminded me of a real patient.

5. I felt that SOPHIE understood what I said.

## Table S7: SOPHIE UI/UX Survey: Feedback Format and Skill Modules

**Feedback Format**

1. I liked the overall format/structure in which the feedback was presented.

2. I liked the way in which the homepage (e.g., first page seen) was presented.

3. I liked the way in which the full feedback (e.g., all feedback when “view full feedback” button is clicked) was presented.

**Skill-Specific Feedback (repeated across 3 modules)**

*Each of the following applies to Empathize, be Explicit, and Empower modules:*

1. I found the “Here is what you did well” section useful.

2. I found the “Here are more opportunities” section useful.

3. I found the “View Suggestion” (e.g., system recommendations on homepage) section useful.

4. I found the “Full transcript with skills highlighted” section useful.

**Additional Module-Specific Feedback**

***be Explicit Module:***

1. I found the “Hedge Words” feedback useful.

2. I found the “Speaking Rate” feedback useful.

3. I found the “Reading Level” feedback useful.

4. Overall, I thought that the way the system quantified “be Explicit” was satisfactory.

***Empower Module:***

1. I found the “Questions Asked” feedback useful.

2. I found the “Open-Ended Questions Asked” useful.

3. I found the “Turn Taking” feedback useful.

4. SOPHIE's responses gave me an opportunity to be empowering.

5. Overall, I thought that the way the system quantified “Empower” was satisfactory.
