# Supplementary material for: Effects of a Generative AI Virtual Patient on Serious Illness Communication Skills: Randomized Controlled Trial
Source: JMIR Med Educ. 2026 Oct 20;12:e93034. doi: 10.2196/93034 (PMC13683997; doi:10.2196/93034)
Supplement: Supplementary file 3 [file mededu-v12-e93034-s002.docx]

Multimedia Appendix

Table of Contents

[Additional Methods	2](#_Toc222517159)

[Dialogue Manager: Schema-Guided + LLM Hybrid	2](#_Toc222517160)

[MVP Framework and 3E Skill Classification	2](#_Toc222517161)

[Standardized Patient Cases	2](#_Toc222517162)

[Cost	3](#_Toc222517163)

[References	3](#_Toc222517164)

# Additional Methods

## MVP Framework and 3E Skill Classification

The **MVP** framework [1] is a structured yet flexible approach for serious illness conversations (SICs) that is supported by three essential communication skills – termed as 3E’s:

1. **Empower** – Ensure patient autonomy, tailor communication, and engage in shared decision-making.
2. **Be Explicit** – Communicate clearly, directly, and concisely to avoid confusion and distress.
3. **Empathize** – Validate and explore emotions, as desired/allowed by the patient, to support patients and families through difficult discussions.

SOPHIE is built on the 3E framework—Empathize, be Explicit, and Empower—which underpins the system’s ability to precisely quantify and deliver actionable feedback on core communication behaviors.

Both for our dialogue generation and feedback, it is important for us to identify if a statement from the user presents one of the 3E skills. Figure 8 in the appendix demonstrates our skill classifier system using a rule-based text classifier and a BERT [2] classifier trained with conversation data from [3]. If either models identify a statement as Empathetic, Empowering, or Explicit, our system tags the user statement with the labeled skill.

SOPHIE’s avatar is a 60-year-old woman with terminal lung cancer. Emotion is modeled using primary states (neutral, sad, angry, happy, surprised, afraid, disgusted). Each has facial animations recorded via motion capture and speech rendered with emotional prosody.

Emotion escalates in response to unacknowledged distress, simulating the impact of communication lapses and promoting behavioral correction.

## Standardized Patient Cases

Participants engaged in one of three randomized simulated cases:

- **Pat Smith:** Optimistic post-chemotherapy patient facing recurrence
- **John/Lois Bell:** Avoidant patient with terminal disease
- **Jack/Jill Cooper:** No treatment options left, discussion of hospice

Cases were developed with URMC oncologists and ACT faculty. We partnered with Professional Communication Simulators to recruit 13 SPs trained in the 3E rubric. Four additional third-party raters evaluated video recordings independently.

## Cost

In this study, the SOPHIE system was running on a local consumer computer with an NVIDIA RTX 3090 GPU. However, it is possible to host the system on a cloud server with 24 × 7 on-demand access, which was implemented after this study was completed. In the most simplistic case, considering the system is running on an Amazon AWS *g4dn.xlarge* EC2 instance, with 100% occupancy, with always a single concurrent meeting running, the approximate cost for our system would be around $1.49 per hour. Hence, the total cost for 26, 30-minute avatar interactions in our study would be approximately $20.

The following is the cost breakdown:

- Cloud compute (1 hour): $0.526 [4]
- Speech-to-text service (30 minutes): $0.5 [5]
- LLM text generation (400K cached, non-cached, output tokens GPT-3.5 Turbo): $0.05 [4]
- Text-to-speech service (27,000 characters spoken): $0.41 [6]

## Feasibility of Running Local Large Language Models

# References

1. Horowitz RK, Hogan LA, Carroll T. Mvp–medical situation, values, and plan: a memorable and useful model for all serious illness conversations. *Journal of Pain and Symptom Management*. 2020;60(5):1059-1065. doi:10.1016/j.jpainsymman.2020.07.022
2. Devlin J, Chang MW, Lee K, Toutanova K. BERT: pre-training of deep bidirectional transformers for language understanding. In: Burstein J, Doran C, Solorio T, eds. Proceedings of the 2019 Conference of the North American Chapter of the Association for Computational Linguistics: Human Language Technologies; June 2019; Minneapolis, MN. Stroudsburg, PA: Association for Computational Linguistics; 2019:4171-4186. doi:10.18653/v1/N19-1423
3. Haut K, Wohn C, Kane B, et al. Validating a virtual human and automated feedback system for training doctor-patient communication skills. In: 2023 11th International Conference on Affective Computing and Intelligent Interaction (ACII). IEEE; 2023:1-8. doi:10.1109/ACII59096.2023.10388213
4. Amazon Web Services. Amazon EC2 On-Demand Pricing. Accessed February 6, 2026. https://aws.amazon.com/ec2/pricing/on-demand/
5. Microsoft Azure. Azure Cognitive Services Speech Pricing. Accessed February 6, 2026. https://azure.microsoft.com/en-us/pricing/details/cognitive-services/speech-services/
6. OpenAI. OpenAI API Pricing. Accessed February 6, 2026. https://openai.com/api/pricing/
